# A method to obtain exact single-step GBLUP for non-genotyped descendants when the genomic relationship matrix of ancestors is not available

**DOI:** 10.1186/s12711-022-00759-x

**Published:** 2022-10-31

**Authors:** Dorian J. Garrick, Rohan L. Fernando

**Affiliations:** 1 Massey University, Ruakura Research Centre, Hamilton, 3240 New Zealand; 2 Iowa State University, 225C Kildee Hall, Ames, IA 50011 USA

## Abstract

**Background:**

Single-step genomic best linear unbiased prediction (GBLUP) involves a joint analysis of individuals with genotype information, and their ancestors, descendants, or contemporaries, without recorded genotypes. Livestock applications typically represent populations with fewer individuals with genotypes relative to the number not genotyped. Most breeding programmes are structured, consisting of a nucleus tier in which selection drives genetic gains that are propagated through descendants that represent parents in multiplier and commercial tiers. In some cases, the genotypes in the nucleus tier are proprietary to a breeding company, and not publicly available for a whole industry analysis. Bayesian inference involves combining a defined description of prior information with new information to generate a posterior distribution that contains all available information on parameters of interest. A natural extension of Bayesian analysis would be to use information from the posterior distribution to define the prior distribution in a subsequent analysis.

**Methods:**

We derive the mixed model equations for inference on breeding values for non genotyped individuals in that subset of the population that is of current interest, using only data on the performance of current individuals and their immediate pedigree, along with prior information defined from the posterior distribution of an external BLUP or single-step GBLUP analysis of the ancestors of the current population.

**Discussion:**

Identical estimates of breeding values and their prediction error covariances for current animals of interest in the multiplier or commercial tier can be obtained without requiring neither the genomic relationship matrix nor genotypes of any of their ancestors in the nucleus tier, as can be obtained from a single analysis using pedigree, performance, and genomic information from all tiers. The Bayesian analysis of the current population does not require explicit information on unselected genotyped animals in the external population.

**Supplementary Information:**

The online version contains supplementary material available at 10.1186/s12711-022-00759-x.

## Background

In the dairy industry, the rate of genetic gain depends on four pathways of selection. Breeding companies control three of these pathways by selecting bulls to breed bulls, cows to breed bulls, and the bulls to breed cows that are made available to the industry. In the genomic era, these selection decisions are typically based on single-step genomic evaluations, using breeding-value [1] single-step genomic best linear unbiased prediction (SS-GBLUP) or marker-effects models [2]. National evaluations that use pedigree-based BLUP (P-BLUP), which does not account for the genomic selection in the three pathways controlled by the breeding companies, will be biased [3]. One solution is to combine all genomic and pedigree information in the national evaluation. However, breeding companies may use proprietary genomic information that they may be reluctant to reveal or make available for the national evaluation, such as panels with different densities, or imputed variants that might include causal mutations.

Martinez and Rothschild [4] presented a recursive approach that is also known as the Kalman filter [5] for sire evaluation that efficiently updates the inverse of the coefficient matrix, estimates of fixed effects, and estimates of breeding values from the most recent evaluation, using only incidence matrices and phenotypes collected since that evaluation. Gianola and Sorensen [6] presented a similar result using a Bayesian updating approach to evaluate only the descendants while incorporating prior information on the merit of ancestors. That Bayesian approach extended results outlined by Gianola [7] and in teaching notes by Quaas [8] and has been used to combine information from external genetic evaluations such as from evaluations in other countries that comprise some of the same ancestors, using the posterior means and covariances of breeding values from the external evaluations [9, 10]. In most applications, however, the posterior covariance matrix is large, its off-diagonals are unknown, and only approximations of its diagonals are available. Furthermore, phenotypes used in the current evaluation may also have been used in the external evaluation. Thus, various approximations are used to combine information from the external evaluations with that from the current data set [11, 12].

In this paper, we present a Bayesian updating approach that does not require the posterior means and covariances for all animals in the external evaluation to obtain the same estimated breeding values for current animals as would be obtained using all data in a single analysis. Suppose the set ${e}$ of animals in the external evaluation are related to the set ${c}$ of current animals only through a subset ${a}$ of animals in set ${e}$ that are immediate ancestors of animals from set ${e}$. In addition, assume that phenotypes used in the current evaluation did not contribute to the external evaluation. Here, we show that estimation of breeding values of the current animals only requires the posterior means and covariances for the animals in subset ${a}$, and not for any of their ancestors or unselected relatives. Similarly, when genomic information is limited to the external evaluation, we show that our approach requires neither the genomic relationship matrix nor genotypes for any of the ancestors to obtain the same results as would be obtained using SS-GBLUP, where all genomic, phenotypic, and pedigree information are combined in a single analysis.

We also show that this result can be derived by first setting up Henderson’s mixed model equations (MME) for the complete pedigree, and then eliminating (i.e. absorbing) the equations for the animals in set ${e}$ that do not belong to subset ${a}$, and all other effects that are unnecessary to the current evaluation. Unlike in the Bayesian approach, such a derivation does not require the assumptions of normality for the breeding values or the residuals.

Our approach readily extends to include fixed effects such as for inbreeding, heterosis or contemporary groups that need to be estimated using data in both the external and current evaluation.

## Methods

Bayesian updating theory is presented here to efficiently combine SS-GBLUP results from an external evaluation with new or unused phenotypic and pedigree data that are available from the current data set. It is assumed that the raw phenotypic and genomic data from the external evaluation are not available, but posterior means and covariances for breeding values of the parents of the animals in the current data set are available. We will first derive formulas to combine results from an external pedigree-based BLUP (P-BLUP) analysis with phenotypic and pedigree information available in the current data set. Then, that derivation will be repeated but extended to use results from an external SS-GBLUP evaluation rather than a P-BLUP evaluation.

### Efficient Bayesian updating using results of a P-BLUP evaluation

#### Bayesian derivation

Let $\mathbf {u}_{\text{e}}$ denote the breeding values of the animals in the external data set and $\mathbf {u}_{\text{c}}$ the breeding values of a set ${c}$ of animals in the current dataset whose data have not contributed to the external P-BLUP analysis. Suppose the conditional distribution of $\mathbf {u}_{\text{c}}$ given $\mathbf {u}_{\text{e}}$ is such that:

$${\text{f}}(\mathbf {u}_{\text{c}}|\mathbf {u}_{\text{e}}) = {\text{f}}(\mathbf {u}_{\text{c}}|\mathbf {u}_{\text{a}}),$$

where $\mathbf {u}_{\text{a}}$, a subset of the elements in $\mathbf {u}_{\text{e}}$, is the vector of breeding values of a set ${a}$ of animals that are the parents of current animals. In the absence of selection, the joint prior for $\mathbf {u}_{\text{a}}$ and $\mathbf {u}_{\text{c}}$ given pedigree information is:

1 $$\left[ \begin{array} {ll} \mathbf {u}_{\text{a}} \\ \mathbf {u}_{\text{c}} \end{array} \right] \sim {\text{N}} \left(\left[\begin{array} {ll} \mathbf {0} \\ \mathbf {0} \end{array} \right], \left[\begin{array}{ll} \mathbf {A}_{\text{aa}} & \mathbf {A}_{\text{ac}} \\ \mathbf {A}_{\text{ca}} & \mathbf {A}_{\text{cc}} \end{array}\right] \upsigma^{2}_{\text{u}} \right).$$

In addition, we assume the current phenotypes can be modeled as:

$$\mathbf {y}_{\text{c}} = \mathbf {X}_{\text{c}}\varvec{\upbeta } + \mathbf {Z}_{\text{c}}\mathbf {u}_{\text{c}} + \mathbf {e}_{\text{c}},$$

where $\varvec{\upbeta }$ is a vector of fixed effects that might include those that are unique to the current dataset and those that are common to the external and current data sets, $\mathbf {X}_{\text{c}}$ has full column rank, implying estimable $\varvec{\upbeta }$, and $\mathbf {e}_{\text{c}}$ is normal with null mean and covariance matrix $\mathbf {I}\upsigma^{2}_{\text{e}}$.

Then, defining the vector of phenotypes used in the external analysis as $\mathbf {y}_{\text{e}}$, under normality of breeding values and residuals, and a flat prior for $\varvec{\upbeta }$, the posterior distribution of $(\varvec{\upbeta }, \mathbf {u}_{\text{a}})|\mathbf {y}_{\text{e}}$ is normal with mean $[\tilde{\varvec{\upbeta }}', \tilde{\mathbf {u}}'_{\text{a}}]'$ and covariance matrix $\mathbf {C}$, which can be estimated from Markov chain Monte Carlo (MCMC) samples drawn from the posterior distribution of $\mathbf {u}_{\text{a}}$ and $\varvec{\upbeta }$. Suppose that conditional on $\varvec{\upbeta }$ and $\mathbf {u}_{\text{c}}$, $\mathbf {y}_{\text{c}}$ is independent of $\mathbf {y}_{\text{e}}$. Then, with a flat prior for $\varvec{\upbeta }$, the posterior distribution of $\mathbf {u}_{\text{a}}$, $\varvec{\upbeta }$, and $\mathbf {u}_{\text{c}}$ given all phenotypes, can be written as:

2 $${\text{f}}(\varvec{\upbeta },\mathbf {u}_{\text{c}},\mathbf {u}_{\text{a}} |\mathbf {y}_{\text{e}},\mathbf {y}_{\text{c}}) \propto {\text{f}}(\varvec{\upbeta },\mathbf {u}_{\text{c}},\mathbf {u}_{\text{a}} ,\mathbf {y}_{\text{e}},\mathbf {y}_{\text{c}})$$

3 $$= {\text{f}}(\mathbf {y}_{\text{c}}|\varvec{\upbeta },\mathbf {u}_{\text{a}},\mathbf {u}_{\text{c}},\mathbf {y}_{\text{e}}) {\text{f}}(\mathbf {u}_{\text{c}}|\varvec{\upbeta },\mathbf {u}_{\text{a}},\mathbf {y}_{\text{e}}) {\text{f}}(\varvec{\upbeta },\mathbf {u}_{\text{a}}|\mathbf {y}_{\text{e}})$$

4 $$= {\text{f}}(\mathbf {y}_{\text{c}}|\varvec{\upbeta },\mathbf {u}_{\text{c}}) {\text{f}}(\mathbf {u}_{\text{c}}|\mathbf {u}_{\text{a}}) {\text{f}}(\varvec{\upbeta },\mathbf {u}_{\text{a}}|\mathbf {y}_{\text{e}})$$

5 $$\propto \exp \left\{-\frac{1}{2}({\text{Q}}_1 + {\text{Q}}_2 + {\text{Q}}_3) \right\},$$

where

$${\text{Q}}_1 = \frac{1}{\upsigma^{2}_{\text{e}}}(\mathbf {y}_{\text{c}} - \mathbf {X}_{\text{c}}\varvec{\upbeta } - \mathbf {Z}_{\text{c}}\mathbf {u}_{\text{c}})'(\mathbf {y}_{\text{c}} - \mathbf {X}_{\text{c}}\varvec{\upbeta } - \mathbf {Z}_{\text{c}}\mathbf {u}_{\text{c}})$$

6 $${\text{Q}}_2 = \frac{1}{\upsigma^{2}_{\text{u}}}(\mathbf {u}_{\text{c}} - \mathbf {A}_{\text{ca}}\mathbf {A}_{\text{aa}}^{-1}\mathbf {u}_{\text{a}})'\mathbf {A}_{{\text{c}}.{\text{a}}}^{-1} (\mathbf {u}_{\text{c}} - \mathbf {A}_{\text{ca}}\mathbf {A}_{\text{aa}}^{-1}\mathbf {u}_{\text{a}})$$

7 $$= \frac{1}{\upsigma^{2}_{\text{u}}}[\mathbf {u}'_{\text{a}} , \mathbf {u}'_{\text{c}}] \left[\begin{array}{cc} \mathbf {A}_{\text{aa}}^{-1}\mathbf {A}_{\text{ac}}\mathbf {A}_{{\text{c}}.{\text{a}}}^{-1}\mathbf {A}_{\text{ca}}\mathbf {A}_{\text{aa}}^{-1} & -\mathbf {A}_{\text{aa}}^{-1}\mathbf {A}_{\text{ac}}\mathbf {A}_{{\text{c}}.{\text{a}}}^{-1} \\ -\mathbf {A}_{{\text{c}}.{\text{a}}}^{-1}\mathbf {A}_{\text{ca}}\mathbf {A}_{\text{aa}}^{-1} & \mathbf {A}_{{\text{c}}.{\text{a}}}^{-1} \end{array}\right] \left[\begin{array}{ll} \mathbf {u}_{\text{a}}\\ \mathbf {u}_{\text{c}} \end{array}\right],$$

$${\text{Q}}_3 = \left[\begin{array}{ll} (\varvec{\upbeta } - \tilde{\varvec{\upbeta }})',(\mathbf {u}_{\text{a}} - \tilde{\mathbf {u}}_{\text{a}})' \end{array}\right] \left[\begin{array}{ll} {\mathbf {C}}^{\upbeta \upbeta } & {\mathbf {C}}^{\upbeta {\text{a}}}\\ {\mathbf {C}}^{{\text{a}}\upbeta } & {\mathbf {C}}^{\text{aa}}\\ \end{array}\right] \left[\begin{array}{ll} (\varvec{\upbeta } - \tilde{\varvec{\upbeta }})\\ (\mathbf {u}_{\text{a}} - \tilde{\mathbf {u}}_{\text{a}}) \end{array}\right],$$

where $$\left[\begin{array}{ll} {\mathbf {C}}^{\upbeta \upbeta } & {\mathbf {C}}^{\upbeta {\text{a}}}\\ {\mathbf {C}}^{{\text{a}}\upbeta } & {\mathbf {C}}^{\text{aa}}\\ \end{array}\right]$$ is the inverse of the matrix ${\mathbf {C}}$ of posterior covariances for $\varvec{\upbeta }$ and $\mathbf {u}_{\text{a}}$ given the external data, and

$$\mathbf {A}_{{\text{c}}.{\text{a}}} = \mathbf {A}_{\text{cc}} - \mathbf {A}_{\text{ca}}\mathbf {A}_{\text{aa}}^{-1}\mathbf {A}_{\text{ac}}.$$

By combining all terms that involve $\mathbf {u}_{\text{a}}$, $\varvec{\upbeta }$, and $\mathbf {u}_{\text{c}}$, the posterior for $\mathbf {u}_{\text{a}}$, $\varvec{\upbeta }$, and $\mathbf {u}_{\text{c}}$ can be written as:

$${\text{f}}(\varvec{\upbeta }, \mathbf {u}_{\text{c}},\mathbf {u}_{\text{a}} |\mathbf {y}_{\text{e}},\mathbf {y}_{\text{c}}) = \exp \left\{-\frac{1}{2}{\text{Q}}\right\},$$

where

8 $$\begin{aligned} {\text{Q}} &= \left[\begin{array}{ll} (\varvec{\upbeta } - \hat{\varvec{\upbeta }})',(\mathbf {u}_{\text{a}} - \hat{\mathbf {u}}_{\text{a}})', (\mathbf {u}_{\text{c}} - \hat{\mathbf {u}}_{\text{c}})' \end{array}\right] \\ & \quad \begin{bmatrix} \frac{1}{\upsigma^{2}_{\text{e}}}\mathbf {X}_{\text{c}}'\mathbf {X}_{\text{c}} + {\mathbf {C}}^{\upbeta \upbeta } & {\mathbf {C}}^{\upbeta {\text{a}}} & \frac{1}{\upsigma^{2}_{\text{e}}}\mathbf {X}_{\text{c}}'\mathbf {Z}_{\text{c}} \\ {\mathbf {C}}^{{\text{a}}\upbeta } & {\mathbf {C}}^{\text{aa}}+\frac{1}{\upsigma^{2}_{\text{u}}}\mathbf {A}_{\text{aa}}^{-1}\mathbf {A}_{\text{ac}}\mathbf {A}_{{\text{c}}.{\text{a}}}^{-1}\mathbf {A}_{\text{ca}}\mathbf {A}_{\text{aa}}^{-1} & -\frac{1}{\upsigma^{2}_{\text{u}}}\mathbf {A}_{\text{aa}}^{-1}\mathbf {A}_{\text{ac}}\mathbf {A}_{{\text{c}}.{\text{a}}}^{-1} \\ \frac{1}{\upsigma^{2}_{\text{e}}}\mathbf {Z}_{\text{c}}'\mathbf {X}_{\text{c}} & -\frac{1}{\upsigma^{2}_{\text{u}}}\mathbf {A}_{{\text{c}}.{\text{a}}}^{-1}\mathbf {A}_{\text{ca}}\mathbf {A}_{\text{aa}}^{-1} & \frac{1}{\upsigma^{2}_{\text{e}}}\mathbf {Z}_{\text{c}}'\mathbf {Z}_{\text{c}}+\frac{1}{\upsigma^{2}_{\text{u}}}\mathbf {A}_{{\text{c}}.{\text{a}}}^{-1} \\ \end{bmatrix} \\ & \quad \left[\begin{array}{ll} (\varvec{\upbeta } - \hat{\varvec{\upbeta }})\\ (\mathbf {u}_{\text{a}} - \hat{\mathbf {u}}_{\text{a}})\\ (\mathbf {u}_c - \hat{\mathbf {u}}_{\text{c}}) \\ \end{array}\right], \end{aligned}$$

which can be recognized as the exponential term of the normal distribution for $\varvec{\upbeta }$, $\mathbf {u}_{\text{a}}$, and $\mathbf {u}_{\text{c}}$, and their means, $\hat{\varvec{\upbeta }}$, $\hat{\mathbf {u}}_{\text{a}}$, and $\hat{\mathbf {u}}_{\text{c}}$, are the solution to:

9 $$\begin{aligned} & \begin{bmatrix} \frac{1}{\upsigma^{2}_{\text{e}}}\mathbf {X}_{\text{c}}'\mathbf {X}_{\text{c}} + {\mathbf {C}}^{\upbeta \upbeta } & {\mathbf {C}}^{\upbeta {\text{a}}} & \frac{1}{\upsigma^{2}_{\text{e}}}\mathbf {X}_{\text{c}}'\mathbf {Z}_{\text{c}} \\ {\mathbf {C}}^{{\text{a}}\upbeta } & {\mathbf {C}}^{\text{aa}}+\frac{1}{\upsigma^{2}_{\text{u}}}\mathbf {A}_{\text{aa}}^{-1}\mathbf {A}_{\text{ac}}\mathbf {A}_{{\text{c}}.{\text{a}}}^{-1}\mathbf {A}_{\text{ca}}\mathbf {A}_{\text{aa}}^{-1} & -\frac{1}{\upsigma^{2}_{\text{u}}}\mathbf {A}_{\text{aa}}^{-1}\mathbf {A}_{\text{ac}}\mathbf {A}_{{\text{c}}.{\text{a}}}^{-1} \\ \frac{1}{\upsigma^{2}_{\text{e}}}\mathbf {Z}_{\text{c}}'\mathbf {X}_{\text{c}} & -\frac{1}{\upsigma^{2}_{\text{u}}}\mathbf {A}_{{\text{c}}.{\text{a}}}^{-1}\mathbf {A}_{\text{ca}}\mathbf {A}_{\text{aa}}^{-1} & \frac{1}{\upsigma^{2}_{\text{e}}}\mathbf {Z}_{\text{c}}'\mathbf {Z}_{\text{c}}+\frac{1}{\upsigma^{2}_{\text{u}}}\mathbf {A}_{{\text{c}}.{\text{a}}}^{-1} \end{bmatrix} \left[\begin{array}{ll} \hat{\varvec{\upbeta }}\\ \hat{\mathbf {u}}_{\text{a}}\\ \hat{\mathbf {u}}_{\text{c}} \\ \end{array}\right] \\ & = \left[ \begin{array}{l} \frac{1}{\upsigma^{2}_{\text{e}}}\mathbf {X}_{\text{c}}'\mathbf {y}_{\text{c}} + {\mathbf {C}}^{\upbeta \upbeta }\tilde{\varvec{\upbeta }} + {\mathbf {C}}^{\upbeta {\text{a}}}\tilde{{\mathbf {u}}}_{\text{a}} \\ {\mathbf {C}}^{{\text{a}}\upbeta }\tilde{\varvec{\upbeta }} + \mathbf {C}^{\text{aa}}\tilde{\mathbf {u}}_{\text{a}} \\ \frac{1}{\upsigma^{2}_{\text{e}}}\mathbf {Z}_{\text{c}}'\mathbf {y}_{\text{c}} \\ \end{array}\right] . \end{aligned}$$

It can be shown that $\mathbf {A}_{{\text{c}}.{\text{a}}}^{-1} = \mathbf {A}^{\text{cc}}$, where a matrix with superscripts represents a submatrix of the inverse of the full matrix (shown below), and that $\mathbf {A}_{\text{ca}}\mathbf {A}_{\text{aa}}^{-1} = -(\mathbf {A}^{\text{cc}})^{-1}\mathbf {A}^{\text{ca}}$ [2]. So, the system of equations that needs to be solved to obtain the posterior means, $\hat{\mathbf {u}}_{\text{a}}$ and $\hat{\mathbf {u}}_{\text{c}}$, given all the information from the external and current data sets, becomes:

10 $$\begin{aligned} & \begin{bmatrix} \frac{1}{\upsigma^{2}_{\text{e}}}\mathbf {X}_{\text{c}}'\mathbf {X}_{\text{c}} + {\mathbf {C}}^{\upbeta \upbeta } & {\mathbf {C}}^{\upbeta {\text{a}}} & \frac{1}{\upsigma^{2}_{\text{e}}}\mathbf {X}_{\text{c}}'\mathbf {Z}_{\text{c}} \\ {\mathbf {C}}^{{\text{a}}\upbeta } & {\mathbf {C}}^{\text{aa}}+ \frac{1}{\upsigma^{2}_{\text{u}}}\mathbf {A}^{\text{ac}}(\mathbf {A}^{\text{cc}})^{-1}\mathbf {A}^{\text{ca}} & \frac{1}{\upsigma^{2}_{\text{u}}}\mathbf {A}^{\text{ac}} \\ \frac{1}{\upsigma^{2}_{\text{e}}}\mathbf {Z}_{\text{c}}'\mathbf {X}_{\text{c}} & \frac{1}{\upsigma^{2}_{\text{u}}}\mathbf {A}^{\text{ca}} & \frac{1}{\upsigma^{2}_{\text{e}}}\mathbf {Z}_{\text{c}}'\mathbf {Z}_{\text{c}}+\frac{1}{\upsigma^{2}_{\text{u}}}\mathbf {A}^{\text{cc}} \end{bmatrix} \left[\begin{array}{ll} \hat{\varvec{\upbeta }}\\ \hat{\mathbf {u}}_{\text{a}} \\ \hat{\mathbf {u}}_{\text{c}} \\ \end{array}\right] \\ & = \left[ \begin{array}{l} \frac{1}{\upsigma^{2}_{\text{e}}}\mathbf {X}_{\text{c}}'\mathbf {y}_{\text{c}} + {\mathbf {C}}^{\upbeta \upbeta }\tilde{\varvec{\upbeta }} + {\mathbf {C}}^{\upbeta {\text{a}}}\tilde{{\mathbf {u}}}_{\text{a}} \\ {\mathbf {C}}^{{\text{a}}\upbeta }\tilde{\varvec{\upbeta }} + \mathbf {C}^{\text{aa}}\tilde{\mathbf {u}}_{\text{a}}\\ \frac{1}{\upsigma^{2}_{\text{e}}}\mathbf {Z}_{\text{c}}'\mathbf {y}_{\text{c}} \\ \end{array}\right] . \end{aligned}$$

Note that these equations only require the posterior mean vectors $\tilde{\varvec{\upbeta }}$ and $\tilde{\mathbf {u}}_{\text{a}}$ and the inverse of their posterior covariance matrix $\mathbf {C}$ from the external evaluation, but the evaluations from Eq. (10) for animals in sets ${a}$ and ${c}$ are identical to those that would be obtained using all information from the external and current data sets in a single analysis.

Setting up these equations in the form shown in Eq. (10) requires computing $\mathbf {A}^{\text{ac}}$ and $\mathbf {A}^{\text{cc}}$, which are submatrices of the inverse of the additive relationship matrix given in Eq. (1):

11 $$\left[\begin{array}{ll} \mathbf {A}_{\text{aa}} & \mathbf {A}_{\text{ac}} \\ \mathbf {A}_{\text{ca}} & \mathbf {A}_{\text{cc}} \end{array}\right]^{-1} = \left[\begin{array}{ll} \mathbf {A}^{\text{aa}} & \mathbf {A}^{\text{ac}} \\ \mathbf {A}^{\text{ca}} & \mathbf {A}^{\text{cc}} \end{array}\right],$$

which can be obtained from those of the inverse of

12 $$\mathbf {A} = \begin{bmatrix} \mathbf {A}_{\text{dd}} & \mathbf {A}_{\text{da}} & \mathbf {A}_{\text{dc}} \\ \mathbf {A}_{\text{ad}} & \mathbf {A}_{\text{aa}} & \mathbf {A}_{\text{ac}} \\ \mathbf {A}_{\text{cd}} & \mathbf {A}_{\text{ca}} & \mathbf {A}_{\text{cc}} \end{bmatrix},$$

which is the additive genetic relationship matrix for the complete pedigree of animals in set ${c}$, with ${d}$ being the set of ancestors of animals from the subset ${e}$ of external animals that do not belong to the set ${a}$. The inverse of $\mathbf {A}$ is known to be very sparse, with non-zero off-diagonals only between mates and between parents and offspring [13]. Thus, let the inverse of this relationship matrix for the full pedigree $\mathbf {A}$ be denoted as $\mathbf {F}^{-1}$, with partitions:

13 $$\mathbf {F}^{-1} = \begin{bmatrix} \mathbf {F}^{\text{dd}} & \mathbf {F}^{\text{da}} & \mathbf {0} \\ \mathbf {F}^{\text{ad}} & \mathbf {F}^{\text{aa}} & \mathbf {F}^{\text{ac}} \\ \mathbf {0} & \mathbf {F}^{\text{ca}} & \mathbf {F}^{\text{cc}} \end{bmatrix},$$

where for example, the submatrix in this inverse that corresponds to $\mathbf {A}_{\text{aa}}$ is denoted as $\mathbf {F}^{\text{aa}}$, which may differ from $\mathbf {A}^{\text{aa}}$ in Eq. (11); furthermore, in Eq. (13) we have recognized that in the inverse of Eq. (12), the submatrix that corresponds to $\mathbf {A}_{\text{dc}}$, and its transpose, are null matrices because animals in ${d}$ do not contain any parents or mates of those in ${c}$. In Appendix 1, we show that two of the submatrices in the right-hand side of Eq. (11) are equal to the corresponding submatrices in the right-hand side of Eq. (13), namely

14 $$\mathbf {A}^{\text{ac}} = \mathbf {F}^{\text{ac}}$$

and

15 $$\mathbf {A}^{\text{cc}} = \mathbf {F}^{\text{cc}}.$$

Thus, the MME in Eq. (10) can be written in a more convenient form as:

16 $$\begin{aligned} & \begin{bmatrix} \frac{1}{\sigma^{2}_{\text{e}}}\mathbf {X}_{\text{c}}'\mathbf {X}_{\text{c}} + {\mathbf {C}}^{\upbeta \upbeta } & {\mathbf {C}}^{\upbeta {\text{a}}} & \frac{1}{\sigma^{2}_{\text{e}}}\mathbf {X}_{\text{c}}'\mathbf {Z}_{\text{e}} \\ {\mathbf {C}}^{{\text{a}}\upbeta } & {\mathbf {C}}^{\text{aa}}+ \frac{1}{\sigma^{2}_{\text{u}}}\mathbf {F}^{\text{ac}}(\mathbf {F}^{\text{cc}})^{-1}\mathbf {F}^{\text{ca}} & \frac{1}{\sigma^{2}_{\text{u}}}\mathbf {F}^{\text{ac}} \\ \frac{1}{\sigma^{2}_{\text{e}}}\mathbf {Z}_{\text{c}}'\mathbf {X}_{\text{c}} & \frac{1}{\sigma^{2}_{\text{u}}}\mathbf {F}^{\text{ca}} & \frac{1}{\sigma^{2}_{\text{e}}}\mathbf {Z}_{\text{c}}'\mathbf {Z}_{\text{c}}+\frac{1}{\sigma^{2}_{\text{u}}}\mathbf {F}^{\text{cc}} \end{bmatrix} \begin{bmatrix} \hat{\varvec{\upbeta }}\\ \hat{\mathbf {u}}_{\text{a}}\\ \hat{\mathbf {u}}_{\text{c}} \\ \end{bmatrix} \\ & = \left[ \begin{array}{l} \frac{1}{\sigma^{2}_{\text{e}}}\mathbf {X}_{\text{c}}'\mathbf {y}_{\text{c}} + {\mathbf {C}}^{\upbeta \upbeta }\tilde{\varvec{\upbeta }} + {\mathbf {C}}^{\upbeta {\text{a}}}\tilde{{\mathbf {u}}}_{\text{a}} \\ {\mathbf {C}}^{{\text{a}}\upbeta }\tilde{\varvec{\upbeta }} + \mathbf {C}^{\text{aa}}\tilde{\mathbf {u}}_{\text{a}} \\ \frac{1}{\sigma^{2}_{\text{e}}}\mathbf {Z}_{\text{c}}'\mathbf {y}_{\text{c}} \\ \end{array}\right] . \end{aligned}$$

These MME involve a quadratic equation comprising the inverse of $\mathbf {F}^{\text{cc}}$, but as shown in Appendix 2,

17 $$\mathbf {F}^{\text{ac}}(\mathbf {F}^{\text{cc}})^{-1}\mathbf {F}^{\text{ca}} = \mathbf {F}^{\text{aa}} - \mathbf {E}^{\text{aa}},$$

where $\mathbf {F}^{\text{aa}}$ is the submatrix in Eq. (13) corresponding to $\mathbf {A}_{\text{aa}}$ from the inverse of $\mathbf {A}$ in Eq. (12), constructed for the complete pedigree of animals in set ${c}$, and submatrix $\mathbf {E}^{\text{aa}}$ in

18 $$\left[\begin{array}{ll} \mathbf {A}_{\text{dd}} & \mathbf {A}_{\text{da}} \\ \mathbf {A}_{\text{ad}} & \mathbf {A}_{\text{aa}} \end{array}\right]^{-1} = \left[\begin{array}{ll} \mathbf {E}^{\text{dd}} & \mathbf {E}^{\text{da}} \\ \mathbf {E}^{\text{ad}} & \mathbf {E}^{\text{aa}} \end{array}\right],$$

is the submatrix corresponding to $\mathbf {A}_{\text{aa}}$ from the inverse of $\mathbf {A}$ constructed for the pedigree used in the external analysis of animals in set ${e}$. Given that both $\mathbf {F}^{\text{aa}}$ and $\mathbf {E}^{\text{aa}}$ are sparse, it is preferable to write Eq. (16) as:

19 $$\begin{aligned} & \begin{bmatrix} \frac{1}{\upsigma^{2}_{\text{e}}}{\mathbf {X}_{\text{c}}^{\prime}} {\mathbf {X}_{\text{c}}} + {\mathbf {C}}^{\upbeta \upbeta } & {\mathbf {C}}^{\upbeta {\text{a}}} & \frac{1}{\upsigma^{2}_{\text{e}}} {\mathbf {X}_{\text{c}}^{\prime}} {\mathbf {Z}_{\text{e}}} \\ {\mathbf {C}}^{{\text{a}}\upbeta } & {\mathbf {C}}^{\text{aa}}+ \frac{1}{\upsigma^{2}_{\text{u}}}(\mathbf {F}^{\text{aa}} - \mathbf {E}^{\text{aa}}) & \frac{1}{\upsigma^{2}_{\text{u}}} {\mathbf {F}^{\text{ac}}} \\ \frac{1}{\upsigma^{2}_{\text{e}}} {\mathbf {Z}_{\text{c}}^{\prime}} {\mathbf {X}_{\text{c}}} & \frac{1}{\upsigma^{2}_{\text{u}}} {\mathbf {F}^{\text{ca}}} & \frac{1}{\upsigma^{2}_{\text{e}}} {\mathbf {Z}_{\text{c}}^{\prime}} {\mathbf {Z}_{\text{c}}}+\frac{1}{\upsigma^{2}_{\text{u}}}{\mathbf {F}^{\text{cc}}} \end{bmatrix} \left[\begin{array}{ll} \hat{\varvec{\upbeta }}\\ {\hat{\mathbf {u}}_{\text{a}}} \\ 
{\hat{\mathbf {u}}_{\text{c}}} \\ \end{array}\right] \\ & \quad = \left[ \begin{array}{l} \frac{1}{\upsigma^{2}_{\text{e}}}{\mathbf {X}_{\text{c}}^{\prime}}{\mathbf {y}_{\text{c}}} + {\mathbf {C}}^{\upbeta \upbeta }\tilde{\varvec{\upbeta }} + {\mathbf {C}}^{\upbeta {\text{a}}} {\tilde{{\mathbf {u}}}_{\text{a}}} \\ {\mathbf {C}}^{{\text{a}}\upbeta } {\tilde{\varvec{\upbeta}}} + {\mathbf {C}^{\text{aa}}} {\tilde{\mathbf {u}}_{\text{a}}} \\ \frac{1}{\upsigma^{2}_{\text{e}}}{\mathbf {Z}_{\text{c}}^{\prime}}{\mathbf {y}_{\text{c}}} \end{array}\right] . \end{aligned}$$

A numerical example is included in Additional file 1 demonstrating that the solution to Eq. (19) for $\hat{\varvec{\upbeta }}$, $\hat{\mathbf {u}}_{\text{a}}$, and $\hat{\mathbf {u}}_{\text{c}}$ are identical to those from a joint analysis of all data.

It should be noted that the matrix difference $\mathbf {F}^{\text{aa}} - \mathbf {E}^{\text{aa}}$ represents the pedigree-based contributions of animals in ${c}$ to their parents in ${a}$ which has been shown in [14] can be constructed in a single pass through the pedigree.

#### Non-Bayesian derivation

Here, we show that the mixed model equations (MME) given in Eq. (16), which were derived using Bayesian arguments, can also be obtained by eliminating the equations for ancestral animals in the set ${d}$ from the usual MME for the full analysis of the complete data in sets ${e}$ and ${c}$. To simplify this presentation, we will ignore all fixed effects in sets ${e}$ and ${c}$ and only present the equations for the breeding values. However, the derivation readily extends to situations where there are fixed effects common to the phenotypic data used in the external and current analyses, in addition to fixed effects that can only be estimated from the external data, and those that can only be estimated from the data contributing to the current analysis.

Consider the MME for joint evaluation of animals from the complete pedigree:

20 $$\begin{aligned} & \begin{bmatrix} \frac{1}{\upsigma^{2}_{\text{e}}}{\mathbf {Z}_{\text{d}}^{\prime}} {\mathbf {Z}_{\text{d}}} + \frac{1}{\upsigma^{2}_{\text{u}}} {\mathbf {F}^{\text{dd}}} & \frac{1}{\upsigma^{2}_{\text{u}}}{\mathbf {F}^{\text{da}}} & {\mathbf {0}} \\ \frac{1}{\upsigma^{2}_{\text{u}}}{\mathbf {F}^{\text{ad}}} & \frac{1}{\upsigma^{2}_{\text{e}}}{\mathbf {Z}_{\text{a}}^{\prime}} {\mathbf {Z}_{\text{a}}} +\frac{1}{\upsigma^{2}_{\text{u}}}{\mathbf {F}^{{\text{aa}}}} & \frac{1}{\upsigma^{2}_{\text{u}}} {\mathbf {F}^{\text{ac}}} \\ {\mathbf {0}} & \frac{1}{\upsigma^{2}_{\text{u}}} {\mathbf {F}^{\text{ca}}} & \frac{1}{\upsigma^{2}_{\text{e}}} {\mathbf {Z}_{\text{c}}^{\prime}}{\mathbf {Z}_{\text{c}}} +\frac{1}{\upsigma^{2}_{\text{u}}} {\mathbf {F}^{\text{cc}}} \end{bmatrix} \left[\begin{array}{ll} {\hat{\mathbf {u}}_{\text{d}}} \\ {\hat{\mathbf {u}}_{\text{a}}} \\ {\hat{\mathbf {u}}_{\text{c}}} \end{array}\right] \\ & \quad = \left[\begin{array}{ll} \frac{1}{\upsigma^{2}_{\text{e}}}{\mathbf {Z}_{\text{d}}^{\prime}} {\mathbf {y}_{\text{d}}} \\ \frac{1}{\upsigma^{2}_{\text{e}}} {\mathbf {Z}_{\text{a}}^{\prime}} {\mathbf {y}_{\text{a}}} \\ \frac{1}{\upsigma^{2}_{\text{e}}} {\mathbf {Z}_{\text{c}}^{\prime}} {\mathbf {y}_{\text{c}}} \end{array}\right]. \end{aligned}$$

To eliminate $\hat{\mathbf {u}}_{\text{d}}$ from these equations, we will use the notation $\mathbf {P} = \frac{1}{\upsigma^{2}_{\text{e}}}\mathbf {Z}_{\text{d}}'\mathbf {Z}_{\text{d}} + \frac{1}{\upsigma^{2}_{\text{u}}}\mathbf {F}^{\text{dd}}$ to represent the leading diagonal block of the MME corresponding to the breeding values of the ancestral animals in ${d}$. Then, the MME with $\hat{\mathbf {u}}_{\text{d}}$ eliminated can be written as:

21 $$\begin{aligned} & \begin{bmatrix} \frac{1}{\upsigma^{2}_{\text{e}}}{\mathbf {Z}_{\text{a}}^{\prime}} {\mathbf {Z}_{\text{a}}} + \frac{1}{\upsigma^{2}_{\text{u}}} {\mathbf {F}^{\text{aa}}} - \frac{1}{\upsigma^{2}_{\text{u}}}{\mathbf {F}^{\text{ad}}} {\mathbf {P}^{-1}} \frac{1}{\upsigma^{2}_{\text{u}}}{\mathbf {F}^{\text{da}}} & \frac{1}{\upsigma^{2}_{\text{u}}} {\mathbf {F}^{\text{ac}}} \\ \frac{1}{\upsigma^{2}_{\text{u}}} {\mathbf {F}^{\text{ca}}} & \frac{1}{\upsigma^{2}_{\text{e}}} {\mathbf {Z}_{\text{c}}^{\prime}} {\mathbf {Z}_{\text{c}}}+\frac{1}{\upsigma^{2}_{\text{u}}} {\mathbf {F}^{\text{cc}}} \end{bmatrix} \left[\begin{array}{ll} {\hat{\mathbf {u}}_{\text{a}}} \\ {\hat{\mathbf {u}}_{\text{c}}} \end{array}\right] \\ & \quad = \left[\begin{array}{cc} \frac{1}{\upsigma^{2}_{\text{e}}} {\mathbf {Z}_{\text{a}}^{\prime}} {\mathbf {y}_{\text{a}}} - \frac{1}{\upsigma^{2}_{\text{u}}}{\mathbf {F}^{\text{ad}}} {\mathbf {P}^{-1}} \frac{1}{\upsigma^{2}_{\text{e}}}{\mathbf {Z}_{\text{d}}^{\prime}} {\mathbf {y}_{\text{d}}} \\ \frac{1}{\upsigma^{2}_{\text{e}}} {\mathbf {Z}_{\text{c}}^{\prime}} {\mathbf {y}_{\text{c}}} \end{array}\right]. \end{aligned}$$

Next, we will show how Eq. (21) can be constructed by combining results from the external evaluation of set ${e}$ with matrices relevant to the evaluation of animals in set ${c}$. The MME for the external evaluation is:

22 $$\begin{aligned} \begin{bmatrix} \frac{1}{\upsigma^{2}_{\text{e}}}\mathbf {Z}_{\text{d}}'\mathbf {Z}_{\text{d}} + \frac{1}{\upsigma^{2}_{\text{u}}}\mathbf {E}^{\text{dd}} & \frac{1}{\upsigma^{2}_{\text{u}}}\mathbf {E}^{\text{da}}\\ \frac{1}{\upsigma^{2}_{\text{u}}}\mathbf {E}^{\text{ad}} & \frac{1}{\upsigma^{2}_{\text{e}}}\mathbf {Z}_{\text{a}}'\mathbf {Z}_{\text{a}}+\frac{1}{\upsigma^{2}_{\text{u}}}\mathbf {E}^{\text{aa}} \end{bmatrix} \left[\begin{array}{ll} \tilde{\mathbf {u}}_{\text{d}}\\ \tilde{\mathbf {u}}_{\text{a}} \end{array}\right] = \left[\begin{array}{ll} \frac{1}{\upsigma^{2}_{\text{e}}}\mathbf {Z}_{\text{d}}'\mathbf {y}_{\text{d}}\\ \frac{1}{\upsigma^{2}_{\text{e}}}\mathbf {Z}_{\text{a}}'\mathbf {y}_{\text{a}} \end{array}\right]. \end{aligned}$$

We show in Appendix 1 that $\mathbf {F}^{\text{ac}}$ and $\mathbf {F}^{\text{cc}}$ in Eq. (13) are identical to $\mathbf {A}^{\text{ac}}$ and $\mathbf {A}^{\text{cc}}$ in Eq. (11). Similarly, it can be shown that $\mathbf {F}^{\text{dd}}$ and $\mathbf {F}^{\text{da}}$ in Eq. (13), which were used in (21), are identical to $\mathbf {E}^{\text{dd}}$ and $\mathbf {E}^{\text{da}}$ in Eq. (18), which were used in Eq. (22). Thus, these MME can be written as:

23 $$\left[\begin{array}{cc} \frac{1}{\upsigma^{2}_{\text{e}}}\mathbf {Z}_{\text{d}}'\mathbf {Z}_{\text{d}} + \frac{1}{\upsigma^{2}_{\text{u}}}\mathbf {F}^{\text{dd}} & \frac{1}{\upsigma^{2}_{\text{u}}}\mathbf {F}^{\text{ad}} \\ \frac{1}{\upsigma^{2}_{\text{u}}}\mathbf {F}^{\text{da}} & \frac{1}{\upsigma^{2}_{\text{e}}}\mathbf {Z}_{\text{a}}'\mathbf {Z}_{\text{a}}+\frac{1}{\upsigma^{2}_{\text{u}}}\mathbf {E}^{\text{aa}} \end{array}\right] \left[\begin{array} {ll} \tilde{\mathbf {u}}_{\text{d}} \\ \tilde{\mathbf {u}}_{\text{a}} \end{array}\right] = \left[\begin{array}{ll} \frac{1}{\upsigma^{2}_{\text{e}}}\mathbf {Z}_{\text{d}}'\mathbf {y}_{\text{d}} \\ \frac{1}{\upsigma^{2}_{\text{e}}}\mathbf {Z}_{\text{a}}'\mathbf {y}_{\text{a}} \end{array}\right],$$

Now, eliminating the equations for $\tilde{\mathbf {u}}_{\text{d}}$ in Eq. (23) gives:

24 $$\left(\frac{1}{\upsigma^{2}_{\text{e}}}\mathbf {Z}_{\text{a}}'\mathbf {Z}_{\text{a}}+\frac{1}{\upsigma^{2}_{\text{u}}}\mathbf {E}^{\text{aa}} - \frac{1}{\upsigma^{2}_{\text{u}}}\mathbf {F}^{\text{ad}}\mathbf {P}^{-1}\frac{1}{\upsigma^{2}_{\text{u}}}\mathbf {F}^{\text{da}}\right)\tilde{\mathbf {u}}_{\text{a}} = \frac{1}{\upsigma^{2}_{\text{e}}}\mathbf {Z}_{\text{a}}'\mathbf {y}_{\text{a}} - \frac{1}{\upsigma^{2}_{\text{u}}}\mathbf {F}^{\text{da}}\mathbf {P}^{-1}\frac{1}{\upsigma^{2}_{\text{e}}}\mathbf {Z}_{\text{d}}'\mathbf {y}_{\text{d}}.$$

Note that $\mathbf {C}_{\text{aa}} = (\frac{1}{\upsigma^{2}_{\text{e}}}\mathbf {Z}_{\text{a}}'\mathbf {Z}_{\text{a}}+\frac{1}{\upsigma^{2}_{\text{u}}}\mathbf {E}^{\text{aa}} - \frac{1}{\upsigma^{2}_{\text{u}}}\mathbf {F}^{\text{ad}}\mathbf {P}^{-1}\frac{1}{\upsigma^{2}_{\text{u}}}\mathbf {F}^{\text{da}})^{-1}$ is the covariance matrix of prediction errors for $\mathbf {u}_{\text{a}}$ from the external analysis. So, using $\tilde{\mathbf {u}}_{\text{a}}$ and $\mathbf {C}_{\text{aa}}$ from the external analysis, Eq. (21) can be written as:

25 $$\left[\begin{array}{cc} \mathbf {C}_{\text{aa}}^{-1} + \frac{1}{\upsigma^{2}_{\text{u}}}\mathbf {F}^{\text{aa}} - \frac{1}{\upsigma^{2}_{\text{u}}}\mathbf {E}^{\text{aa}} & \frac{1}{\upsigma^{2}_{\text{u}}}\mathbf {F}^{\text{ac}}\\ \frac{1}{\upsigma^{2}_{\text{u}}}\mathbf {F}^{\text{ca}} & \frac{1}{\upsigma^{2}_{\text{e}}}\mathbf {Z}_{\text{c}}'\mathbf {Z}_{\text{c}}+\frac{1}{\upsigma^{2}_{\text{u}}}\mathbf {F}^{\text{cc}} \end{array}\right] \left[\begin{array}{ll} \hat{\mathbf {u}}_{\text{a}} \\ \hat{\mathbf {u}}_{\text{c}} \end{array}\right] = \left[\begin{array}{ll} \mathbf {C}_{\text{aa}}^{-1}\tilde{\mathbf {u}}_{\text{a}} \\ \frac{1}{\upsigma^{2}_{\text{e}}}\mathbf {Z}_{\text{c}}'\mathbf {y}_{\text{c}} 
\end{array}\right].$$

These MME are identical to those derived by the Bayesian approach in Eq. (19), except for the components corresponding to the fixed effects, which were ignored in this derivation.

### Extension to SS-GBLUP

In SS-GBLUP, following [1], the joint prior for $\mathbf {u}_{\text{a}}$ and $\mathbf {u}_{\text{c}}$ given pedigree and marker information is taken to be:

$$\left[\begin{array}{ll} \mathbf {u}_{\text{a}} \\ \mathbf {u}_{\text{c}} \end{array}\right] \sim {\text{N}} \left(\left[\begin{array}{ll} \mathbf {0} \\ \mathbf {0} \end{array}\right], \left[\begin{array}{ll} \mathbf {H}_{\text{aa}} & \mathbf {H}_{\text{ac}} \\ \mathbf {H}_{\text{ca}} & \mathbf {H}_{\text{cc}} \end{array}\right] \upsigma^{2}_{\text{u}} \right).$$

Then, the MME for combining the external information for SS-GBLUP becomes:

26 $$\begin{aligned} & \begin{bmatrix} \frac{1}{\upsigma^{2}_{\text{e}}}{\mathbf {X}_{\text{c}}^{\prime}} {\mathbf {X}_{\text{c}}} + {\mathbf {C}}^{\upbeta \upbeta} & {\mathbf {C}}^{\upbeta{\text{a}}} & \frac{1}{\upsigma^{2}_{\text{e}}} {\mathbf {X}_{\text{c}}^{\prime}} {\mathbf {Z}_{\text{c}}} \\ {\mathbf {C}}^{{\text{a}}\upbeta } & {\mathbf {C}}^{\text{aa}} + \frac{1}{\upsigma^{2}_{\text{u}}} {\mathbf {H}^{\text{ac}}} (\mathbf {H}^{\text{cc}})^{-1} {\mathbf {H}^{\text{ca}}} & \frac{1}{\upsigma^{2}_{\text{u}}}{\mathbf {H}^{\text{ac}}} \\ \frac{1}{\upsigma^{2}_{\text{e}}} {\mathbf {Z}_{\text{c}}^{\prime}} {\mathbf {X}_{\text{c}}} & \frac{1}{\upsigma^{2}_{\text{u}}}{\mathbf {H}^{\text{ca}}} & \frac{1}{\upsigma^{2}_{\text{e}}} {\mathbf {Z}_{\text{c}}^{\prime}} {\mathbf {Z}_{\text{c}}} +\frac{1}{\upsigma^{2}_{\text{u}}}{\mathbf {H}^{\text{cc}}} \end{bmatrix} \left[\begin{array}{ll} {\hat{\varvec{\upbeta }}_{\text{c}}} \\ {\hat{\mathbf {u}}_{\text{a}}} \\ {\hat{\mathbf {u}}_{\text{c}}} \\ \end{array}\right] \\ & \quad = \left[ \begin{array}{l} \frac{1}{\upsigma^{2}_{\text{e}}} {\mathbf {X}_{\text{c}}^{\prime}} {\mathbf {y}_{\text{c}}} + {\mathbf {C}}^{\upbeta \upbeta } {\tilde{\varvec{\upbeta}}} + {\mathbf {C}}^{\upbeta {\text{a}}}\tilde{{\mathbf {u}}}_{\text{a}} \\ {\mathbf {C}}^{{\text{a}}\upbeta }\tilde{\varvec{\upbeta}} + {\mathbf {C}^{\text{aa}}} {\tilde{\mathbf{u}}_{\text{a}}} \\ \frac{1}{\upsigma^{2}_{\text{e}}}{\mathbf {Z}_{\text{c}}^{\prime}} {\mathbf {y}_{\text{c}}} \\ \end{array}\right] , \end{aligned}$$

where $\tilde{\varvec{\upbeta }}$ and $\tilde{\mathbf {u}}_{\text{a}}$ are the posterior means of $\varvec{\upbeta }$ and $\mathbf {u}_{\text{a}}$ from the external SS-GBLUP analysis and ${\mathbf {C}}^{\upbeta \upbeta }$, ${\mathbf {C}}^{\upbeta{\text{a}}}$, ${\mathbf{C}}^{\text{a}\upbeta}$, and ${\mathbf {C}}^{\text{aa}}$ are their posterior covariance matrices from the external SS-GBLUP analysis, which are algebraically equal to the relevant blocks of the inverse of the left-hand side matrix. Given that genomic information is limited to the external data set, the only dense part of $\mathbf {H}^{-1}$ will be the block corresponding to the animals in set ${a}$. So, $\mathbf {H}^{\text{ac}} = \mathbf {A}^{\text{ac}}$ = $\mathbf {F}^{\text{ac}}$, and $\mathbf {H}^{\text{cc}} = \mathbf {A}^{\text{cc}}= \mathbf {F}^{\text{cc}}$, and the MME become:

27 $$\begin{aligned} & \begin{bmatrix} \frac{1}{\upsigma^{2}_{\text{e}}}{\mathbf {X}_{\text{c}}^{\prime}} {\mathbf {X}_{\text{c}}} + {\mathbf {C}}^{\upbeta \upbeta} & {\mathbf {C}}^{\upbeta {\text{a}}} & \frac{1}{\upsigma^{2}_{\text{e}}} {\mathbf {X}_{\text{c}}^{\prime}} {\mathbf {Z}_{\text{c}}} \\ {\mathbf {C}}^{{\text{a}}\upbeta } & {\mathbf {C}}^{\text{aa}} + \frac{1}{\upsigma^{2}_{\text{u}}} {\mathbf {H}^{\text{ac}}} (\mathbf {H}^{\text{cc}})^{-1} {\mathbf {H}^{\text{ca}}} & \frac{1}{\upsigma^{2}_{\text{u}}}{\mathbf {F}^{\text{ac}}} \\ \frac{1} {\upsigma^{2}_{\text{e}}} {\mathbf {Z}_{\text{c}}^{\prime}} {\mathbf {X}_{\text{c}}} & \frac{1}{\upsigma^{2}_{\text{u}}}{\mathbf {F}^{{\text{ca}}}} & \frac{1}{\upsigma^{2}_{\text{e}}} {\mathbf {Z}_{\text{c}}^{\prime}} {\mathbf {Z}_{\text{c}}} +\frac{1}{\upsigma^{2}_{\text{u}}}{\mathbf {F}^{\text{cc}}} \\ \end{bmatrix} \left[\begin{array}{ll} {\hat{\varvec{\upbeta}}_{\text{c}}} \\ {\hat{\mathbf{u}}_{\text{a}}} \\ {\hat{\mathbf {u}}_{\text{c}}} \\ \end{array}\right] \\ & \quad = \left[ \begin{array}{ll} \frac{1}{\upsigma^{2}_{\text{e}}} {\mathbf {X}_{\text{c}}^{\prime}} {\mathbf {y}_{\text{c}}} + {\mathbf {C}}^{\upbeta \upbeta } {\tilde{\varvec{\upbeta}}} + {\mathbf {C}}^{\upbeta {\text{a}}} {\tilde{{\mathbf{u}}}_{\text{a}}} \\ {\mathbf {C}}^{{\text{a}}\upbeta }\tilde{\varvec{\upbeta }} + {\mathbf {C}^{\text{aa}}} {\tilde{\mathbf {u}}_{\text{a}}} \\ \frac{1}{\upsigma^{2}_{\text{e}}}{\mathbf {Z}_{\text{c}}^{\prime}} {\mathbf {y}_{\text{c}}} \\ \end{array}\right] . \end{aligned}$$

The posterior mean vectors and covariance matrices in these MME can be estimated from MCMC samples for $\varvec{\upbeta }$ and $\mathbf {u}_{\text{a}}$ drawn from their posterior in the external analysis. The identities given in Eqs. (14), (15) and (17) can be used to write the MME as:

28 $$\begin{aligned} & \begin{bmatrix} \frac{1}{\upsigma^{2}_{\text{e}}}{\mathbf {X}_{\text{c}}^{\prime}} {\mathbf {X}_{\text{c}}} + {\mathbf {C}}^{\upbeta \upbeta } & {\mathbf {C}}^{\upbeta {\text{a}}} & \frac{1}{\upsigma^{2}_{\text{e}}} {\mathbf {X}_{\text{c}}^{\prime}} {\mathbf {Z}_{\text{c}}} \\ {\mathbf {C}}^{{\text{a}}\upbeta } & {\mathbf {C}}^{\text{aa}} + \frac{1}{\upsigma^{2}_{\text{u}}}(\mathbf {F}^{\text{aa}} - {\mathbf {E}^{\text{cc}}}) & \frac{1}{\upsigma^{2}_{\text{u}}} {\mathbf {F}^{\text{ac}}} \\ \frac{1}{\upsigma^{2}_{\text{e}}} {\mathbf {Z}_{\text{c}}^{\prime}} {\mathbf {X}_{\text{c}}} & \frac{1}{\upsigma^{2}_{\text{u}}} {\mathbf {F}^{\text{ca}}} & \frac{1}{\upsigma^{2}_{\text{e}}} {\mathbf {Z}_{\text{c}}^{\prime}} {\mathbf {Z}_{\text{c}}} +\frac{1}{\upsigma^{2}_{\text{u}}} {\mathbf {F}^{\text{cc}}} \end{bmatrix} \left[\begin{array}{ll} {\hat{\varvec{\upbeta }}_{\text{c}}} \\ {\hat{\mathbf {u}}_{\text{a}}} \\ {\hat{\mathbf {u}}_{\text{c}}} \\ \end{array}\right] \\ & \quad = \left[ \begin{array}{ll} \frac{1}{\upsigma^{2}_{\text{e}}}{\mathbf {X}_{\text{c}}^{\prime}} {\mathbf {y}_{\text{c}}} + {\mathbf {C}}^{\upbeta \upbeta} {\tilde{\varvec{\upbeta}}} + {\mathbf {C}}^{\upbeta {\text{a}}} {\tilde{{\mathbf {u}}}_{\text{a}}} \\ {\mathbf {C}}^{{\text{a}}\upbeta } {\tilde{\varvec{\upbeta}}} + {\mathbf {C}^{\text{aa}}} {\tilde{\mathbf {u}}_{\text{a}}} \\ \frac{1}{\upsigma^{2}_{\text{e}}}{\mathbf {Z}_{\text{c}}^{\prime}} {\mathbf {y}_{\text{c}}} \\ \end{array}\right] . \end{aligned}$$

## Practical implementation and discussion

A full evaluation would use pedigree, phenotypic, and genomic data on all individuals in the population. The equalities derived in this paper show that a current evaluation using only a subset of the pedigree and phenotypes, and without any individual genomic information, can provide identical predictions of random effects and corresponding reliabilities on current animals. The posterior means and variances for parents of the current animals must be available from an external evaluation to be used as priors in the evaluation. In circumstances where some fixed effects such as for inbreeding, heterosis, or contemporary effects, are relevant to phenotypes in both the external and current analyses, then the posterior information from those effects would also need to be available from the external evaluation, as previously described by Henderson [15]. We omit some of the details for handling these fixed effects common to the two analyses in the description below, but include that case in the example we describe in Additional file 1.

Operationally, the following steps would be required to efficiently implement our approach.

### External analysis

1. Run a so-called external evaluation, for example using MCMC for P-BLUP or SS-GBLUP, so as to calculate a vector of EBV and their corresponding matrix of prediction error variance-covariances (PEV) from an analysis that excludes the current animals.
2. Store the EBV = $\tilde{\mathbf {u}}_{\text{a}}$ and PEV = $\mathbf {C}_{\text{aa}}$ from the external analysis for those animals that are immediate ancestors of the current animals. In the event that fixed effects such as heterosis are common to the external and current analyses, the fixed effects solutions $\tilde{\mathbf {\upbeta }}$ and the corresponding blocks $\mathbf {C}_{\upbeta \upbeta }$, $\mathbf {C}_{\upbeta {\text{a}}}$, and $\mathbf {C}_{{\text{a}}\upbeta }$, also need to be stored.
3. Extract just that partition of the inverse numerator relationship matrix from the external analysis ($\mathbf {E}^{\text{aa}}$) that corresponds to the immediate ancestors of the current animals for which the PEV were stored.

### Current analysis

1. Compute the inverse numerator relationship matrix for the full pedigree including all current and external animals, then obtain the submatrix partitions corresponding to the ancestors from the external analysis and the current animals ($\mathbf {F}^{\text{aa}}, \mathbf {F}^{\text{ac}},\mathbf {F}^{\text{ca}},\; \text {and}\; \mathbf {F}^{\text{cc}}$), but omitting the partitions corresponding to the distant ancestors ($\mathbf {F}^{\text{dd}}, \mathbf {F}^{\text{da}}, \; \text {and} \; \mathbf {F}^{\text{ad}}$).
2. Form the least squares equations corresponding to the mixed model equations for the current analysis, including equations for random effects corresponding to only the current and direct ancestors, but not the distant ancestors from the external analysis.
3. Augment the submatrix partitions of the random effects with the corresponding partitions of the inverse numerator relationship matrices ($\mathbf {F}^{\text{aa}}, \mathbf {F}^{\text{ac}},\mathbf {F}^{\text{ca}},\; \text {and}\; \mathbf {F}^{\text{cc}}$) multiplied by the scalar inverse genetic variance.
4. If no fixed effects are common to the two analyses, invert the PEV for ancestors obtained from the external analysis ($\mathbf {C}_{\text{aa}}^{-1}$), otherwise invert the matrix $\mathbf {C}$ of posterior covariances for $\varvec{\upbeta }$ and $\mathbf {u_{\text{a}}}$ given the external data to obtain $\mathbf {C}^{\upbeta \upbeta }$, $\mathbf {C}^{\upbeta {\text{a}}}$, $\mathbf {C}^{\text{a}\upbeta }$, and $\mathbf {C}^{\text{aa}}$.
5. Augment the left-hand side matrix by adding ($\mathbf {C}^{\text{aa}}$) and subtracting the corresponding submatrix partition of the inverse of the numerator relationship matrix for the ancestors based on the pedigree used in the external analysis multiplied by the scalar inverse genetic variance. Recall that subtraction of the latter matrix can be avoided if $\mathbf {F}^{\text{aa}}$ − $\mathbf {E}^{\text{aa}}$ is directly computed [14] and used in place of $\mathbf {F}^{\text{aa}}$ in step 1 of the current analysis.
6. If fixed effects are common to the external and current analyses, further augment the relevant left hand side matrix block of $\mathbf {X_{\text{c}}'X_{\text{c}}}$ with $\mathbf {C}^{\upbeta \upbeta }$ and the offdiagonal blocks that relate fixed effects to breeding values with $\mathbf {C}^{\text{a}\upbeta }$ and $\mathbf {C}^{\upbeta {\text{a}}}$.
7. Augment the right-hand side by adding the inverse PEV times the EBV ($\mathbf {C}^{\text{aa}}\tilde{\mathbf {u}}_{\text{a}}$) for the external animals that need to be included in the current analysis.
8. If fixed effects are common to the external and current analyses, further augment the relevant submatrices on the right hand side with $\mathbf {C}^{\text{a}\upbeta }\tilde{\mathbf {\upbeta }}$, $\mathbf {C}^{\upbeta {\text{a}}}\tilde{\mathbf {u}}_{\text{a}}$, and $\mathbf {C}^{\upbeta \upbeta }\tilde{\mathbf {\upbeta }}.$
9. Solve the resulting equations, for example by MCMC.

The order of the equations in the external analysis plus the order of the equations in the current analysis must at least slightly exceed the order of the equations in a full combined analysis, because equations for any parents of animals in the current analysis that are in the external analysis will be present in both analyses, as well as any fixed effects common to both analyses. Nevertheless, the overall computing effort can be reduced by separate external and current analyses, as the analyses can occur at different frequencies, and the memory requirements are reduced in separate analyses compared to a combined analysis.

The exact analysis described requires a PEV matrix from the external analysis. The use of MCMC samplers to obtain PEV is becoming increasingly common in many applications, including those with millions of animals, such as in the multibreed national dairy cattle evaluation in New Zealand (https://www.dairynz.co.nz/animal/animal-evaluation/), the Pan American Hereford beef cattle evaluation run by the American Hereford Association (https://hereford.org/genetics/breed-improvement/epd-trends/), and the international multibreed beef cattle evaluation run by International Genetic Solutions (https://www.internationalgeneticsolutions.com/site/). Nevertheless, many organisations still rely on preconditioned conjugate gradient (PCG) solvers and use approximations for the diagonal elements of the PEV matrix. The use of approximations will not enable the exact same predictions to be obtained as from a single analysis, as prediction error covariances are influenced by data structure, such as the distribution of progeny across contemporary groups, as well as by pedigree relationships. In circumstances where only a small number of animals in the external evaluation represent the parents in the current analysis, the PEV matrix could be reconstructed using PCG one row/column at a time by setting the right-hand side matrix to null, except for a single element of unity corresponding to an animal in set ${a}$.

The structure of the data that allows the simplifications shown in this approach will not necessarily exist when arbitrarily partitioning a dataset into an external and a current analysis. In particular, the derivations took advantage of the inverse numerator relationship matrix having a null off-diagonal partition for the animals in the current analysis relative to distant ancestors in the external analysis, as in Eq. (13). Nevertheless, there are circumstances when such data structures already exist. One such circumstance is when the external evaluation includes the nucleus tier of a breeding population and the current analysis includes a commercial tier that sources one or both sexes of parents from the nucleus, but does not contribute any of these descendants to the nucleus. This represents a form of a closed nucleus breeding scheme with respect to the animals that are comprised in the current analysis.

Application of the approach may extend to situations where the genomic relationship matrix of ancestors is available, but the current analysis might use posterior information from the external analysis simply to reduce computational effort. The frequencies with which the external and the current analysis may be undertaken could be quite different. For example, in a pig breeding enterprise, the external analysis might include all purebred individuals in the herds that comprise the nucleus tier and the current analysis might include all purebred and crossbred animals that were born in and comprise the multiplier tier, along with their immediate purebred ancestors that were sourced from the nucleus. In this case, the frequencies of either of the evaluations would be dictated by the frequencies with which meaningful new data become available in the corresponding tiers.

In dairy and other animal industries, there is often considerable variation in the quality of data collected on farm. Information nucleus herds, herds that include bull dams, dedicated progeny testing herds, or herds used for measuring novel traits might be included in an external analysis. Large-scale commercial herds that can be characterised with poorer recording practices could define the animals used in the current analysis. An extreme example might comprise an external single-step analysis followed by a current analysis that includes only a single herd, as had been previously defined for P-BLUP [8, 16]. The current analysis could even be conducted on-farm, in real time, and would require only limited computing effort. This might be useful for herds that use systems such as inline milk meters that by nature will have less reliable predictions of milk composition than those obtained by central laboratories using state of the art Fourier transform mid-infrared spectroscopy. Such herds would obtain evaluations that benefited from the accurate information available from the external evaluation on the sires they used, yet these herds likely add little real information to improve the accuracy of ancestors that already have high reliability EBV.

In circumstances where genomic information or selection practices applied in the nucleus tiers may be proprietary, the managers of those herds may be reluctant to share genotypes or phenotypes. This might be because the generation of high quality phenotypic data and a suitably scaled genomic training population provides a barrier to entry for competitors, or it might be because proprietary loci such as causal mutations that are being retained as trade secrets represent a competitive advantage that would be eroded by sharing. In these circumstances, the approach developed in this paper shows that only the posterior means and covariances of the immediate ancestors of the animals in the current analysis need to be publicly shared. Neither the details of the selection intensities applied to genomic screening of young sires, nor the identities, pedigrees or genotypes of those selection candidates that did not get selected for subsequent use, need to be shared. All the information that accounts for pre-selection bias is contained within the posterior information derived from the external analysis.

The method described can be applied to more complex models than those used in this paper. That is, the concept of using information from the posterior distribution from one analysis as prior information in a subsequent analysis readily extends to marker effects models, multiple-trait models, maternal effects models, random regression models, threshold models, etc.

The approach is, however, not easily extended to general circumstances where the current dataset includes genotyped as well as non genotyped individuals. In that situation it is not sufficient to use the posterior distribution of marker effects from the external analysis as prior information in the current analysis. This is because in single-step analyses marker covariates for non-genotyped individuals are implicitly or explicitly imputed by regression of non genotyped animals on the genotypes of all pedigree relatives.

## Conclusion

We have demonstrated using a Bayesian derivation assuming normality, and confirmed from a mixed model approach absorbing effects of no immediate use, that recursive updating is possible without the need for access to all data. We show that inferences identical to those made from a complete analysis of all pedigree, performance, and genomic information can be made by combining pedigree and performance information for current animals with prior information obtained from an external evaluation that need not extend beyond that of the parents in the current analysis. Information regarding any genomic or other preselection that was characterised in the external analysis is not directly required in the current analysis, as all that information is conveyed in the posterior information from the immediate ancestors that are represented in the current analysis. That posterior information comprises the prior information in the current analysis. In spite of the current popularity of single-step methods that reanalyze all information for every evaluation, we believe there is utility in analyses that carry relevant information from the posterior information that is obtained from one analysis forward as prior information in a subsequent analysis, without recourse to all the pedigree, performance, or genomic information that was used in the previous analysis.

### Supplementary Information

**Additional file 1.** Numerical example of Bayesian updating of a P-BLUP evaluation. This example shows that Bayesian updating of an external evaluation with the current data yields identical results to those from a joint analysis of the data from the external and the current evaluations. In this example, the posterior mean vectors and covariance matrices that are needed for the Bayesian updating analysis are obtained directly from the solutions and the inverse of the coefficient matrix for the MME of the external analysis, rather than from MCMC samples. The PDF file shows the Julia script and the results from running that script. The Jupyter Notebook containing the Julia script will require a Jupyter Notebook application to run or modify the script.

## Appendix 1

Here, we show that $\mathbf {A}^{\text{ac}}$ and $\mathbf {A}^{\text{cc}}$, which are submatrices of Eq. (11), are identical to $\mathbf {F}^{\text{ac}}$ and $\mathbf {F}^{\text{cc}}$ from Eq. (13). To show this, consider the inverse of the positive-definite matrix, $\mathbf {V}$, partitioned as:

29 $$\left[\begin{array}{ll} \mathbf {V}_{11} & \mathbf {V}_{12} \\ \mathbf {V}_{21} & \mathbf {V}_{22} \end{array}\right]^{-1} = \left[\begin{array}{ll} \mathbf {V}^{11} & \mathbf {V}^{12} \\ \mathbf {V}^{21} &\mathbf {V}^{22} \end{array}\right] .$$

Partitioned matrix results [17] can be used to show that:

$$\mathbf {V}^{22} = (\mathbf {V}_{22} - \mathbf {V}_{21}\mathbf {V}_{11}^{-1}\mathbf {V}_{12})^{-1},$$

and similarly

$$\mathbf {V}_{22} = (\mathbf {V}^{22} - \mathbf {V}^{21}(\mathbf {V}^{11})^{-1}\mathbf {V}^{12})^{-1}.$$

Then, it follows that:

30 $$\mathbf {V}_{22}^{-1} = \mathbf {V}^{22} - \mathbf {V}^{21}(\mathbf {V}^{11})^{-1}\mathbf {V}^{12}.$$

So, by taking $\mathbf {V}^{11}=\mathbf {F}^{\text{dd}}$, $\mathbf {V}^{12} = [\mathbf {F}^{\text{da}},\mathbf {0}]$, and

$$\mathbf {V}^{22} = \begin{bmatrix} \mathbf {F}^{\text{aa}} & \mathbf {F}^{\text{ac}} \\ \mathbf {F}^{\text{ca}} & \mathbf {F}^{\text{cc}} \end{bmatrix},$$

the inverse in Eq. (11) can be written as:

31 $$\left[\begin{array}{ll} \mathbf {A}_{\text{aa}} & \mathbf {A}_{\text{ac}} \\ \mathbf {A}_{\text{ca}} & \mathbf {A}_{\text{cc}} \end{array}\right]^{-1} = \left[\begin{array}{cc} \mathbf {F}^{\text{aa}} - \mathbf {F}^{\text{ad}}(\mathbf {F}^{\text{dd}})^{-1}\mathbf {F}^{\text{da}} & \mathbf {F}^{\text{ac}} \\ \mathbf {F}^{\text{ca}} & \mathbf {F}^{\text{cc}} \end{array}\right]$$

32 $$= \begin{bmatrix} \mathbf {A}^{\text{aa}} & \mathbf {A}^{\text{ac}} \\ \mathbf {A}^{\text{ca}} & \mathbf {A}^{\text{cc}} \end{bmatrix},$$

and $\mathbf {A}^{\text{ac}} = \mathbf {F}^{\text{ac}}$ and $\mathbf {A}^{\text{cc}} = \mathbf {F}^{\text{cc}}$, which are sparse and can be computed using Henderson’s efficient algorithm [13] with the complete pedigree for the animals in the sets ${e}$ and ${c}$.

## Appendix 2

Here we show that $\mathbf {F}^{\text{aa}} - \mathbf {E}^{\text{aa}} = \mathbf {A}^{\text{ac}}(\mathbf {A}^{\text{cc}})^{-1}\mathbf {A}^{\text{ca}}$. Given the notation in Eq. (29), partitioned matrix results [17] can be used to show that:

$$\mathbf {V}_{11} = (\mathbf {V}^{11} - \mathbf {V}^{12}(\mathbf {V}^{22})^{-1}\mathbf {V}^{21})^{-1},$$

and

33 $$\mathbf {V}_{11}^{-1} = \mathbf {V}^{11} - \mathbf {V}^{12}(\mathbf {V}^{22})^{-1}\mathbf {V}^{21}.$$

Now, taking $\mathbf {V}^{11}$ = $$\left[\begin{array}{ll} \mathbf {F}^{\text{dd}} & \mathbf {F}^{\text{da}} \\ \mathbf {F}^{\text{ad}} & \mathbf {F}^{\text{aa}} \end{array}\right]$$, $\mathbf {V}^{21} = [\mathbf {0}, \mathbf {F}^{\text{ca}}]$, and $\mathbf {V}^{22} = \mathbf {F}^{\text{cc}}$,

the inverse in Eq. (18) becomes:

34 $$\left[\begin{array}{ll} \mathbf {A}_{\text{dd}} & \mathbf {A}_{\text{da}} \\ \mathbf {A}_{\text{ad}} & \mathbf {A}_{\text{aa}} \end{array}\right]^{-1} = \left[\begin{array}{cc} \mathbf {F}^{\text{dd}} & \mathbf {F}^{\text{da}} \\ \mathbf {F}^{\text{ad}} & \mathbf {F}^{\text{aa}} - \mathbf {F}^{\text{ac}}(\mathbf {F}^{\text{cc}})^{-1}\mathbf {F}^{\text{ca}} \end{array}\right]$$

35 $$= \left[\begin{array}{ll} \mathbf {E}^{\text{dd}} & \mathbf {E}^{\text{da}} \\ \mathbf {E}^{\text{ad}} & \mathbf {E}^{\text{aa}} \end{array}\right],$$

which shows that $\mathbf {E}^{\text{aa}} = \mathbf {F}^{\text{aa}} - \mathbf {F}^{\text{ac}}(\mathbf {F}^{\text{cc}})^{-1}\mathbf {F}^{\text{ca}}$, and

$$\mathbf {F}^{\text{aa}} - \mathbf {E}^{\text{aa}} = \mathbf {F}^{\text{ac}}(\mathbf {F}^{\text{cc}})^{-1}\mathbf {F}^{\text{ca}}.$$
