# Supplementary material for: A method to obtain exact single-step GBLUP for non-genotyped descendants when the genomic relationship matrix of ancestors is not available
Source: Genet Sel Evol. 2022 Oct 31;54:72. doi: 10.1186/s12711-022-00759-x (PMC9620661; doi:10.1186/s12711-022-00759-x)
Supplement: Supplementary file 1 — Additional file 1. Numerical example of Bayesian updating of a P-BLUP evaluation. This example shows that Bayesian updating of an external evaluation with the current data yields identical results to those from a joint analysis of the data from the external and the current evaluations. In this example, the posterior mean vectors and covariance matrices that are needed for the Bayesian updating analysis are obtained directly from the solutions and the inverse of the coefficient matrix for the MME of the external analysis, rather than from MCMC samples. The PDF file shows the Julia script and the results from running that script. The Jupyter Notebook containing the Julia script will require a Jupyter Notebook application to run or modify the script. [file 12711_2022_759_MOESM1_ESM.zip › exampleWithFixedEffects.pdf]

Numerical Example with Fixed Effects

In [1]:
```
using XSim
using DataFrames
using CSV
using JWAS
using SparseArrays
using LinearAlgebra
using Tables
```

Simulation of Data

In [2]:
```
CLEAR()
build_genome(n_chr=10,n_loci=100);
```

```
--------- Genome Summary ---------
Number of Chromosome  : 10

Chromosome Length (cM):
[100.0, 100.0, 100.0, 100.0, 100.0, 100.0, 100.0, 100.0, 100.0, 100.0]

Number of Loci        : 1000
[100, 100, 100, 100, 100, 100, 100, 100, 100, 100]

Genotyping Error      : 0.0
Mutation Rate         : 0.0
```

In [3]:
```
build_phenome([1000]);
```

```
--------- Phenome Summary ---------
Number of Traits      : 1
Heritability (h2)     : [0.5]
Number of QTLs        : [1000;;]
Genetic (Co)variance
 1.0
Residual (Co)variance
 1.0
QTL Effects (Only first 30 markers are shown)
  0.05
  0.008
 -0.06
  0.026
 -0.022
  0.064
 -0.023
  0.018
  0.048
  0.043
  0.041
  0.002
 -0.088
  0.04
  0.008
  0.065
  0.043
  0.05
 -0.015
  0.027
 -0.028
 -0.025
  0.005
 -0.012
  0.01
 -0.033
 -0.003
  0.048
  0.016
 -0.031
```

In [4]:

```
function samplePed(ped::Matrix)
    animalVec = GLOBAL("animals")
    N = size(animalVec,1)
    n = size(ped,1)
    animals = Array{Animal}(undef, n)
    for i = 1:n
        indID = ped[i,1]
        sireID = ped[i,2]
        damID  = ped[i,3]
        if sireID == 0
            if indID <= N
                animal = animalVec[indID]
            else
                animal = Animal(Animal(),Animal())
            end
        else
            if sireID > size(animalVec,1)
                println("sireID = $sireID is not valid")
                return 0
            end
            sire = animalVec[sireID]
            if damID > size(animalVec,1)
                println("damID = $damID is not valid")
                return 0
            end
            dam = animalVec[damID]
            animal = Animal(sire,dam)
        end
        animals[i] = animal
    end
    return Cohort(animals)
end
```

Out[4]:

```
samplePed (generic function with 1 method)
```

In [5]:
```
founders = [collect(1:8) repeat([0 0],inner=(8,1))]

eliteParents = [
9  1 2
10 3 4
11 5 6
12 7 8
]

Np = 1

progeny =
[
collect(1:Np).+12      repeat([9 10],inner=(Np,1))
collect(1:Np).+(12+Np) repeat([11 12],inner=(Np,1))
]

myPed = [
    founders
    eliteParents
    progeny
]

CSV.write("smallPed.csv",DataFrame(myPed),header=false)
```

Out[5]:
```
"smallPed.csv"
```

In [6]:
```
ped = Matrix(CSV.read("smallPed.csv",DataFrame,header=false))
animals = samplePed(ped);
```

Simulated Pedigree

In [7]:
```
ped
```

Out[7]:
```
14×3 Matrix{Int64}:
  1   0   0
  2   0   0
  3   0   0
  4   0   0
  5   0   0
  6   0   0
  7   0   0
  8   0   0
  9   1   2
 10   3   4
 11   5   6
 12   7   8
 13   9  10
 14  11  12
```

Simulated Phenotypes

In [8]:
```
yAll = get_phenotypes(animals);
```

In [9]:
```
N  = 12 + 2Np
```

Out[9]:
```
14
```

Incidence Matrix for Fixed Effect of Sex

In [10]:
```
sex = repeat([1,2],Int(N/2));
```

In [11]:
```
Xsex = zeros(N,2)
for i=1:N
    j = sex[i]
    Xsex[i,j] = 1.0
end
Xsex
```

Out[11]:
```
14×2 Matrix{Float64}:
 1.0  0.0
 0.0  1.0
 1.0  0.0
 0.0  1.0
 1.0  0.0
 0.0  1.0
 1.0  0.0
 0.0  1.0
 1.0  0.0
 0.0  1.0
 1.0  0.0
 0.0  1.0
 1.0  0.0
 0.0  1.0
```

Additive Relationship Matrix and its Inverse

In [12]:
```
Ped  = JWAS.PedModule.get_pedigree("smallPed.csv");
temp = Matrix(JWAS.PedModule.AInverse(Ped));
indx = [Ped.idMap[string(i)].seqID for i in 1:N]
Ai   = temp[indx,indx]
A = inv(Ai)
round.(A,digits=2)
```

```
The delimiter in smallPed.csv is ','.
Pedigree information:
#individuals: 14
#sires:       6
#dams:        6
#founders:    8
```

Out[12]:

```
14×14 Matrix{Float64}:
 1.0   0.0   0.0   0.0   0.0   0.0   …  0.5   0.0  0.0  -0.0   0.25  -0.0
 0.0   1.0   0.0   0.0   0.0   0.0      0.5   0.0  0.0  -0.0   0.25  -0.0
 0.0   0.0   1.0   0.0   0.0   0.0      0.0   0.5  0.0  -0.0   0.25  -0.0
 0.0   0.0   0.0   1.0   0.0   0.0      0.0   0.5  0.0  -0.0   0.25  -0.0
 0.0   0.0   0.0   0.0   1.0   0.0      0.0  -0.0  0.5   0.0  -0.0    0.25
 0.0   0.0   0.0   0.0   0.0   1.0   …  0.0  -0.0  0.5   0.0  -0.0    0.25
 0.0   0.0   0.0   0.0   0.0   0.0      0.0  -0.0  0.0   0.5  -0.0    0.25
 0.0   0.0   0.0   0.0   0.0   0.0      0.0  -0.0  0.0   0.5  -0.0    0.25
 0.5   0.5   0.0   0.0   0.0   0.0      1.0   0.0  0.0  -0.0   0.5   -0.0
 0.0   0.0   0.5   0.5   0.0   0.0      0.0   1.0  0.0  -0.0   0.5   -0.0
 0.0   0.0   0.0   0.0   0.5   0.5   …  0.0   0.0  1.0   0.0  -0.0    0.5
 0.0   0.0   0.0   0.0   0.0   0.0      0.0   0.0  0.0   1.0  -0.0    0.5
 0.25  0.25  0.25  0.25  0.0   0.0      0.5   0.5  0.0   0.0   1.0   -0.0
 0.0   0.0   0.0   0.0   0.25  0.25     0.0   0.0  0.5   0.5   0.0    1.0
```

In [13]:

```
Ai
```

Out[13]:

```
14×14 Matrix{Float64}:
  1.5   0.5   0.0   0.0   0.0   0.0  …  -1.0   0.0   0.0   0.0   0.0   0.0
  0.5   1.5   0.0   0.0   0.0   0.0     -1.0   0.0   0.0   0.0   0.0   0.0
  0.0   0.0   1.5   0.5   0.0   0.0      0.0  -1.0   0.0   0.0   0.0   0.0
  0.0   0.0   0.5   1.5   0.0   0.0      0.0  -1.0   0.0   0.0   0.0   0.0
  0.0   0.0   0.0   0.0   1.5   0.5      0.0   0.0  -1.0   0.0   0.0   0.0
  0.0   0.0   0.0   0.0   0.5   1.5  …   0.0   0.0  -1.0   0.0   0.0   0.0
  0.0   0.0   0.0   0.0   0.0   0.0      0.0   0.0   0.0  -1.0   0.0   0.0
  0.0   0.0   0.0   0.0   0.0   0.0      0.0   0.0   0.0  -1.0   0.0   0.0
 -1.0  -1.0   0.0   0.0   0.0   0.0      2.5   0.5   0.0   0.0  -1.0   0.0
  0.0   0.0  -1.0  -1.0   0.0   0.0      0.5   2.5   0.0   0.0  -1.0   0.0
  0.0   0.0   0.0   0.0  -1.0  -1.0  …   0.0   0.0   2.5   0.5   0.0  -1.0
  0.0   0.0   0.0   0.0   0.0   0.0      0.0   0.0   0.5   2.5   0.0  -1.0
  0.0   0.0   0.0   0.0   0.0   0.0     -1.0  -1.0   0.0   0.0   2.0   0.0
  0.0   0.0   0.0   0.0   0.0   0.0      0.0   0.0  -1.0  -1.0   0.0   2.0
```

Joint Analysis

In [14]:
```
#X = [Xcg Xsex]
Xj = Xsex
Zj = I(N)
y = yAll

mme = [Xj'Xj    Xj'Zj
       Zj'Xj    Zj'Zj + Ai]
rhs = [Xj'y
       Zj'y]
Ginv = pinv(mme)
solJoint = Ginv*rhs;
```

External Analysis

In [15]:
```
X = Xsex[1:12,:]
Z = I(12)
y = yAll[1:12]
Ei = inv(A[1:12,1:12])

mme = [X'X    X'Z
       Z'X    Z'Z + Ei]

rhs = [X'y
       Z'y]
GiExt = inv(mme)
solExt = GiExt*rhs;
```

Bayesian Updating

In [16]:
```
B = Ai[9:N,9:N]                                  # From inverse of full pedigree
B[1:4,1:4] = B[1:4,1:4] - Ei[9:12,9:12];  # using formula 17 to modify subma
```

In [17]:
```
# equations numbers from external analysis for fixed effects and for breedir
r =[1;2;collect(11:14)]
C = inv(GiExt[r,r])  # inverse of posterior covariance matrix for fixed effe
X = Xsex[13:end,:]
Z = [zeros(2Np,4) I(2Np)]
y = yAll[13:end]
mme = [X'X    X'Z
       Z'X    Z'Z + B]
mme[1:6,1:6] = mme[1:6,1:6] + C
rhs = [X'y; Z'y]
rhs[1:6] = rhs[1:6] + C*solExt[r]
solUpdate = inv(mme)*rhs;
```

Solutions for Fixed Effects from Joint and Bayesian Updating Analyses

In [18]:
```
[solJoint[1:2]  solUpdate[1:2]]
```

Out[18]:
```
2×2 Matrix{Float64}:
  0.334132   0.334132
 -0.225502  -0.225502
```

Solutions for Breeding Values from Joint and Bayesian Updating Analyses

In [19]:
```
[solJoint[11:end]  solUpdate[3:end]]
```

Out[19]:
```
6×2 Matrix{Float64}:
 -0.466214   -0.466214
  0.0922963   0.0922963
 -0.113158   -0.113158
 -0.371218   -0.371218
 -0.252155   -0.252155
 -0.276687   -0.276687
```
